# Sex differences in neural responses to reward and the influences of individual reward and punishment sensitivity

**DOI:** 10.1186/s12868-021-00618-3

**Published:** 2021-02-27

**Authors:** Isha Dhingra, Sheng Zhang, Simon Zhornitsky, Wuyi Wang, Thang M. Le, Chiang-Shan R. Li

**Affiliations:** 1 Department of Psychiatry, Yale University School of Medicine, New Haven, CT 06520 USA; 2 Department of Neuroscience, Yale University School of Medicine, New Haven, CT 06520 USA; 3 Interdepartmental Neuroscience Program, Yale University, New Haven, CT 06520 USA; 4 Connecticut Mental Health Center S112, 34 Park Street, New Haven, CT 06519-1109 USA

**Keywords:** Gender, Reward, Punishment, Individual difference, fMRI, MIDT

## Abstract

**Background:**

Men and women show differences in sensitivity to reward and punishment, which may impact behavior in health and disease. However, the neural bases of these sex differences remain under-investigated. Here, by combining functional magnetic resonance imaging (fMRI) and a variant of the Monetary Incentive Delay Task (MIDT), we examined sex differences in the neural responses to wins and losses and how individual reward and punishment sensitivity modulates these regional activities.

**Methods:**

Thirty-sex men and 27 women participated in the fMRI study. We assessed sensitivity to punishment (SP) and sensitivity to reward (SR) with the Sensitivity to Punishment and Sensitivity to Reward Questionnaire (SPSRQ). In the MIDT, participants pressed a button to collect reward ($1, 1¢, or nil), with the reaction time window titrated across trials so participants achieved a success rate of approximately 67%. We processed the Imaging data with published routines and evaluated the results with a corrected threshold.

**Results:**

Women showed higher SP score than men and men showed higher SR score than women. Men relative to women showed higher response to the receipt of dollar or cent reward in bilateral orbitofrontal and visual cortex. Men as compared to women also showed higher response to dollar loss in bilateral orbitofrontal cortex. Further, in whole-brain regressions, women relative to men demonstrated more significant modulation by SP in the neural responses to wins and larger wins, and the sex differences were confirmed by slope tests.

**Conclusions:**

Together, men showed higher SR and neural sensitivity to both wins, large or small, and losses than women. Individual differences in SP were associated with diminished neural responses to wins and larger wins in women only. These findings highlight how men and women may differ in reward-related brain activations in the MIDT and add to the imaging literature of sex differences in cognitive and affective functions.

## Background

### Reward processing in health and illness

Reward-directed behavior is fundamental to survival and well-being [1]. We strive to obtain primary (food, water, sex) and secondary (money, social approval) rewards [2, 3]. Reward-seeking behavior engages neural circuits central to motivation and learning [4, 5]. Numerous studies have identified the ventral tegmental area, ventral striatum (VS), orbitofrontal cortex (OFC), and anterior cingulate cortex as key regions for reward processing [6–10]. These structures integrate motivational and cognitive processes to support reward-seeking behavior [4, 8, 11]. Further, individuals vary in how they respond to reward-related contingencies [12], and men appear to be more sensitive to reward whereas women are more sensitive to punishment [13, 14].

Many neuropsychiatric conditions implicate motivation deficits and reward processing dysfunction. For instance, anhedonia and compulsive drug seeking represent core symptoms of major depression and substance use disorders (SUDs), respectively, and implicate dysfunction of the reward circuitry [15, 16]. Studies have consistently found reduced striatal response to reward anticipation and feedback [17] and impaired learning of reward contingencies [18] in patients with depression. The neural deficits correlate positively with anhedonia and negatively with treatment outcome [4, 19, 20]. Chronic pain is frequently comorbid with depression and associated with deficits in reward responses [21]. Substance misuse alters the salience of natural reinforcers and compromises self-control of immediate gratification, perpetuating drug seeking [4, 22–24]. Adolescents exposed prenatally to maternal cigarette smoking are observed to have a weaker bilateral VS response to reward anticipation [25]. Importantly, many of these neuropsychiatric conditions demonstrate sex differences in their clinical profiles and etiological processes, with, for instance, women more vulnerable to depression and men to SUDs. Thus, it is important to better understand how men and women process reward differently.

### Sex differences in reward and punishment processing

Biological, including hormonal, and socio-cultural factors may all account for sex differences in reward sensitivity and reward-directed behavior [26, 27]. Indeed, the literature is mixed regarding sex differences in reward-related behavior and the neural processes underlying such differences. For example, some studies showed that women relative to men were better at delaying gratification [28, 29], but others showed no sex differences [30]. A study in rodents reported no sex difference in reward-guided associative learning across multiple paradigms. However, females appeared to be faster in learning to avoid punishment and, after learning, exhibited higher sensitivity than males to probabilistic punishment but less sensitive when punishment could be avoided with certainty [27]. Women compared to men tended to pick decks with lower frequency of punishment on a gambling task [31]. In a study combining electroencephalography (EEG) and a guessing task with both reward and punishment feedback, boys showed lower feedback-related negativity (FRN) and less behavioral changes following punishment [32]. Further, girls but not boys demonstrated FRN to punishment in correlation with a reward sensitivity trait. In another EEG study of an incentive delay task, boys relative to girls showed less stimulus-preceding negativity in anticipation of punishment and greater feedback P3 to monetary than social reward [33]. These findings are consistent with a literature of higher female sensitivity to loss, punishment or other negative feedback [27, 34, 35].

In contrast, visual sexual stimuli activated the reward system in both sexes whereas the VS was involved in men but not women in supporting the distractor effects of the stimuli on line orientation judgment [36]. Adolescent boys relative to girls showed greater VS activation during reward processing in a risky decision making task, made a higher percentage of risky selections, and self-reported greater motivation to earn money than girls [37]. Studying the perceived value of monetary vs. social rewards using a monetary/social incentive delay task, another group reported activation of a wider mesolimbic network in response to anticipation of monetary reward in men and in anticipation of both monetary and social reward in women, in accord with their reaction time performance [38]. In a recent work of a reward go/no-go task, men exhibited greater arousal in response to “go” action (predominating monetary wins), and the arousal better predicted go success rate, as compared to women [39]. On the other hand, women were better than men in learning from positive (but not negative) feedback in a probabilistic selection task [31]. In another study of the Human Connectome Project data, women relative to men showed more suppression of the default mode circuit and higher activation of the dorsal attention circuit during exposure to both reward and punishment, suggesting enhanced saliency of both reward and punishment in women [40]. Thus, sex differences in reward-related processing appear to depend on the nature of rewarding stimuli and behavioral contingency.

### The effects of reward and punishment sensitivity on reward-related processing

In addition to sex, individuals may also vary in reward-related behavioral and neural processes because of distinct reward and punishment sensitivity. Sensitivity to reward scores correlated positively with reactivity to erotic pictures in the left OFC, left insula, and right VS [41]. In a study of reinforcement learning, individual differences in reward sensitivity were positively associated with bilateral VS activation during receipt of reward, while differences in punishment sensitivity were negatively associated with left dorsal striatal activity during loss anticipation and with right lateral OFC activation during loss feedback [42]. Another study examined three groups of people, each with low, medium, and high reward sensitivity, in a working memory task with sub- and supra-liminal stimuli. Individuals with medium reward sensitivity improved performance with high reward in both subliminal and supraliminal conditions, whereas the effect of reward was stronger in the supraliminal than subliminal condition for those with high reward sensitivity scores [43]. The latter findings highlighted complex effects of individual reward sensitivity on cognitive performance incentivized by monetary reward. Investigators have also associated sensitivity to punishment with higher insula activity during feedback of social loss in a social incentive delay task in people with subthreshold depression [44]. On the other hand, despite its wide use in the imaging literature, no studies of the monetary incentive delay task (MIDT) have examined how neural responses to monetary wins or losses may vary with individual sensitivity to reward or to punishment.

## Methods

### Aim, design and setting of the study

The present study aims to characterize sex differences in cerebral responses to reward anticipation and feedback and whether women and men differ in the influences of individual reward and punishment sensitivity on these neural processes. On the basis of the literature, we broadly hypothesized higher SR in men relative to women and reward circuit responses in correlation with SR in both men and women. We would also explore how SP modulated neural response to wins and losses and potential sex differences in SP modulation. To these ends we contrasted men and women in regional responses to reward anticipation and feedback in neurotypical populations. We performed linear regression to identify how these regional responses may vary according to individual SR and SP and examined the sex differences in these correlations with slope tests. Understanding sex differences in reward processing would provide information for translational studies to examine sex-specific neural markers of clinical conditions that implicate reward processing dysfunction.

### Subjects and assessments

Sixty-three healthy adults (27 women; 22–55 or 37 ± 11, mean ± SD, years of age) participated in this study. All subjects were healthy with no current use of prescription medications. None reported a history of head injury or neurological illness. Other exclusion criteria included current or past Axis I Disorders including dependence on a psychoactive substance, according to DSM-IV. All participants were evaluated with the Sensitivity to Reward and Sensitivity to Punishment Questionnaire (SPSRQ) [45]. The SPSRQ contains 48 yes–no items, half concerning the scale for behavioral impulsivity/responsiveness to reward and half concerning the scale for behavioral avoidance in response to potentially adverse consequences. Scores were obtained by totaling the number of yes-answers in each scale, with a higher sub-score each indicating higher sensitivity to reward (SR) and sensitivity to punishment (SP). Table 1 summarizes subject characteristics.

**Table 1:** Subject characteristics

	Men (n = 36)	Women (n = 27)	p value
Age (years)	38.3 ± 10.4	34.3 ± 10.2	0.15
Education (years)	15.5 ± 3.7	15.1 ± 2.7	0.62
SR	9.9 ± 5.1	9.0 ± 3.6	0.04*
SP	7.3 ± 4.7	10.0 ± 5.6	0.04*

*SR* sensitivity to reward, *SP* sensitivity to punishment

^*^p < 0.05, two-sample t test

The Human Investigation Committee at Yale University School of Medicine approved the study and all subjects gave written informed consent prior to participation.

### Monetary incentive delay task (MIDT)

**Fig. 1:**
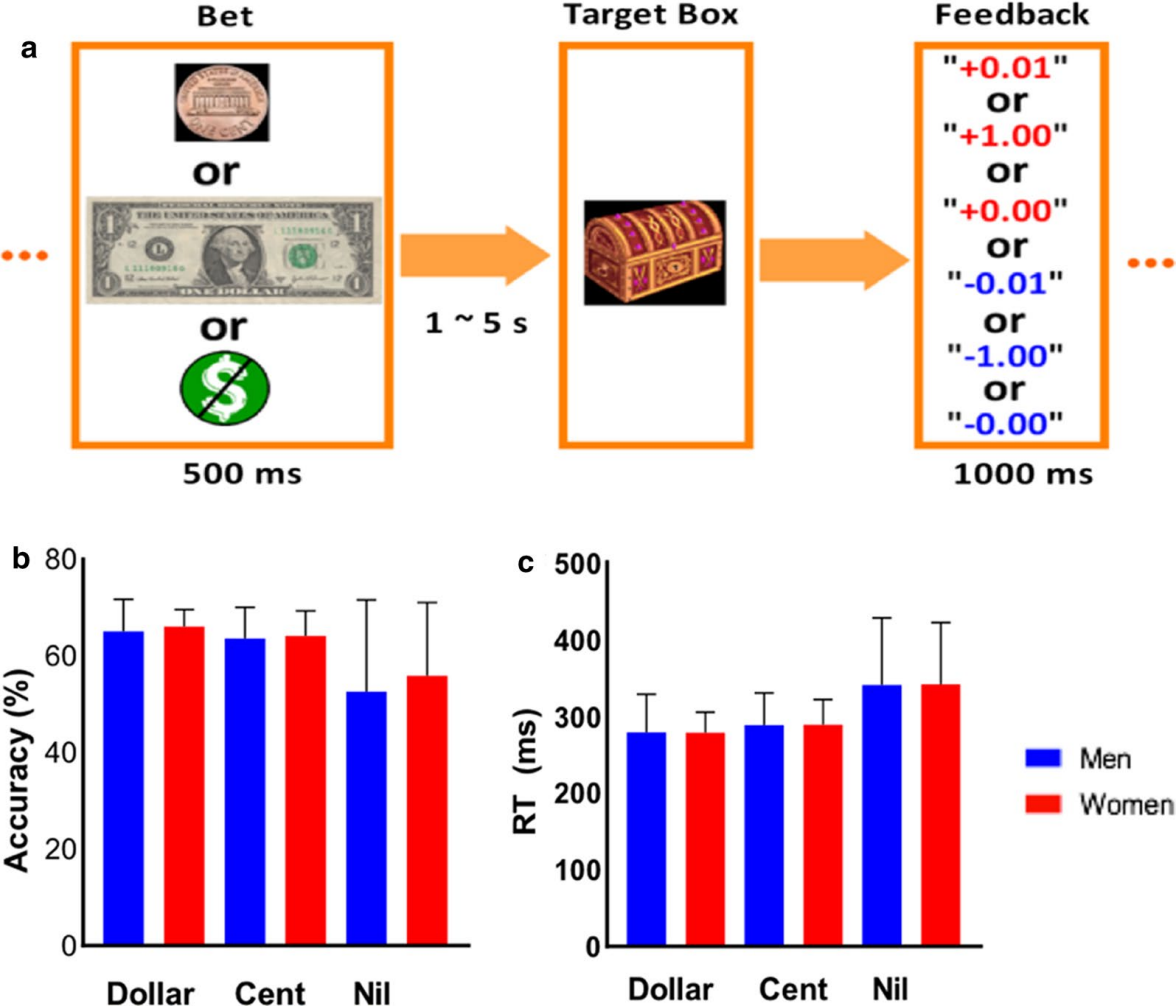
Behavioral paradigm and performance. a Monetary incentive delay task: A bet (a dollar, a cent, or no money) appeared at the beginning of each trial. After a randomized interval between 1 and 5 s, a target box appeared on the screen, then disappeared after a short period (response window). Subjects were told to press a button as quickly as possible to collect the money in the target box (win) before it disappeared. Otherwise, subjects lost the bet, with the amount deducted from their total winnings. A premature button-press prior to the appearance of the target box terminated the trial, and similarly resulted in loss. A feedback window was shown on the screen after each trial to indicate the amount of money won or lost. b Accuracy rate and c RT of dollar, cent and no money (nil) trials (mean ± SD) for men and women

In the MIDT (Fig. 1a), a bet (a dollar, a cent, or no money, randomly intermixed) appeared on the screen at the beginning of each trial. After a randomized fore-period between 1 and 5 s (uniform distribution), a target box was shown for a short period (response window, see below). Subjects were told to press a button as quickly as possible to collect the money (win) before the target box disappeared. An accurate trial was defined by a button press before disappearance of the target box. Otherwise, subjects would lose the bet, with the amount deducted from the total win. A premature button press prior to the appearance of the target box terminated the trial, and similarly resulted in loss. Feedback was shown on the screen after each trial to indicate the amount of money won or lost. Approximately 42% of all trials were dollar trials, 42% were cent trials, and “no money” constituted the remaining trials. The inter-trial-interval was 1.5 s. The response window started at 300 ms, and was stair-cased for each trial type (dollar/cent/no money) separately; for instance, if the subject succeeded at two successive dollar trials, the window decreased by 30 ms, making it more difficult to win again; conversely, if a subject failed for two successive trials, the response window increased by 30 ms, making it easier to win. We anticipated that the subjects would win in approximately 67% each for dollar and cent trials. Each subject completed two 10-min runs of the task. Across subjects, there were 184 ± 4 (mean ± SD) trials in a study.

### Imaging protocol, data preprocessing, and modeling

Brain images were collected using multiband imaging with a 3-T MR scanner (Siemens Trio, Erlangen, Germany). Conventional T1-weighted spin echo sagittal anatomical images were acquired for slice localization. Anatomical 3D magnetization-prepared rapid acquisition with gradient echo (MPRAGE) image were next obtained with spin echo imaging in the axial plane parallel to the anterior commissure-posterior commissure (AC–PC) line with repetition time (TR) = 1900 ms, echo time (TE) = 2.52 ms, bandwidth = 170 Hz/pixel, field of view = 250 × 250 mm, matrix = 256 × 256, 176 slices with slice thickness = 1 mm and no gap. Functional, blood oxygen level-dependent (BOLD) signals were then acquired with a single-shot gradient echo echoplanar imaging (EPI) sequence. Fifty-one axial slices parallel to the AC–PC line covering the whole brain were acquired with TR = 1000 ms, TE = 30 ms, bandwidth = 2290 Hz/pixel, flip angle = 62°, field of view = 210 × 210 mm, matrix = 84 × 84, 51 slices with slice thickness = 2.5 mm and no gap, multiband acceleration factor = 3.

Data were analyzed with Statistical Parametric Mapping (SPM8). Individual subjects’ images were corrected for motion and slice timing. A mean functional image volume was constructed for individual subjects per run from the realigned image volumes, co-registered with the high-resolution structural image, and segmented for normalization with affine registration followed by nonlinear transformation [46, 47]. The normalization parameters determined for the structure volume were applied to the corresponding functional volumes for individual subjects. The images were then smoothed with a Gaussian kernel of 8 mm at Full Width at Half Maximum.

We examined event-related BOLD signals in a general linear model, where we distinguished the cue onset of dollar, cent, and “no money” or nil trials, and the feedback onset of dollar win, dollar loss, cent win, cent loss, and nil. A statistical analytical design was constructed for each individual subject, with the onsets of individual trials convolved with a canonical hemodynamic response function (HRF) and with the temporal derivatives of the canonical HRF and entered as regressors in the model [48]. Realignment parameters in all six dimensions were also entered in the model. Serial autocorrelation caused by aliased cardiovascular and respiratory effects was corrected by a first-degree autoregressive model. The GLM estimated the component of variance explained by each of the regressors.

In group level or random effects analyses, we employed one-sample t tests to examine whole-brain responses to individual contrasts (see below) in men and women combined and two-sample t tests to examine sex differences in these contrasts with age and RT differences, as specified by the contrasts, as covariates. To investigate the neural correlates of SR and SP, we conducted whole-brain linear regressions of these contrasts on SR and SP, with age and RT differences, as specified by the contrasts, as covariates for men and women combined as well as separately. For sex-specific findings, we defined the functional clusters identified from linear regressions in men and women alone as regions of interest, extracted the β estimates for all subjects, and performed slope tests to examine sex differences in the correlations [49]. Note that the slope test for sex difference does not represent “double-dipping” or circular analysis, as the slope tests may confirm or refute sex differences [39, 69].

All models were evaluated with a threshold combining voxel p < 0.001, uncorrected and cluster p < 0.05 family-wise error (FWE) corrected, following current reporting standards. Voxels with peak activity were indicated with Montreal Neurological Institute (MNI) coordinates. We have also deposited the unthresholded maps of all contrasts on Neurovalut (https://identifiers.org/neurovault.collection:9057).

## Results

### Behavioral performance

Figure 1b, c show the accuracy rate (AR) and reaction time (RT) of dollar, cent, and nil trials. For both men and women, the ARs were close to 67%, suggesting the success of the staircase procedure. For men, the AR ranged from 40 to 71% for dollar, 41 to 71% for cent, and 41 to 70% for nil trials. For women, the AR ranged from 55 to 71% for dollar, 43 to 69% for cent and 49 to 69% for nil trials. There were no significant sex differences in the AR of dollar (t_56_ = − 0.76; p = 0.45), cent (t_61_ =  − 0.37; p = 0.70), or nil (t_61_ =  − 0.73; p = 0.47) trials or in the RT of dollar (t_56_ = 0.05; p = 0.96), cent (t_61_ =  − 0.04; p = 0.97), or nil (t_58_ = 0.04; p = 0.97) trials.

**Fig. 2:**
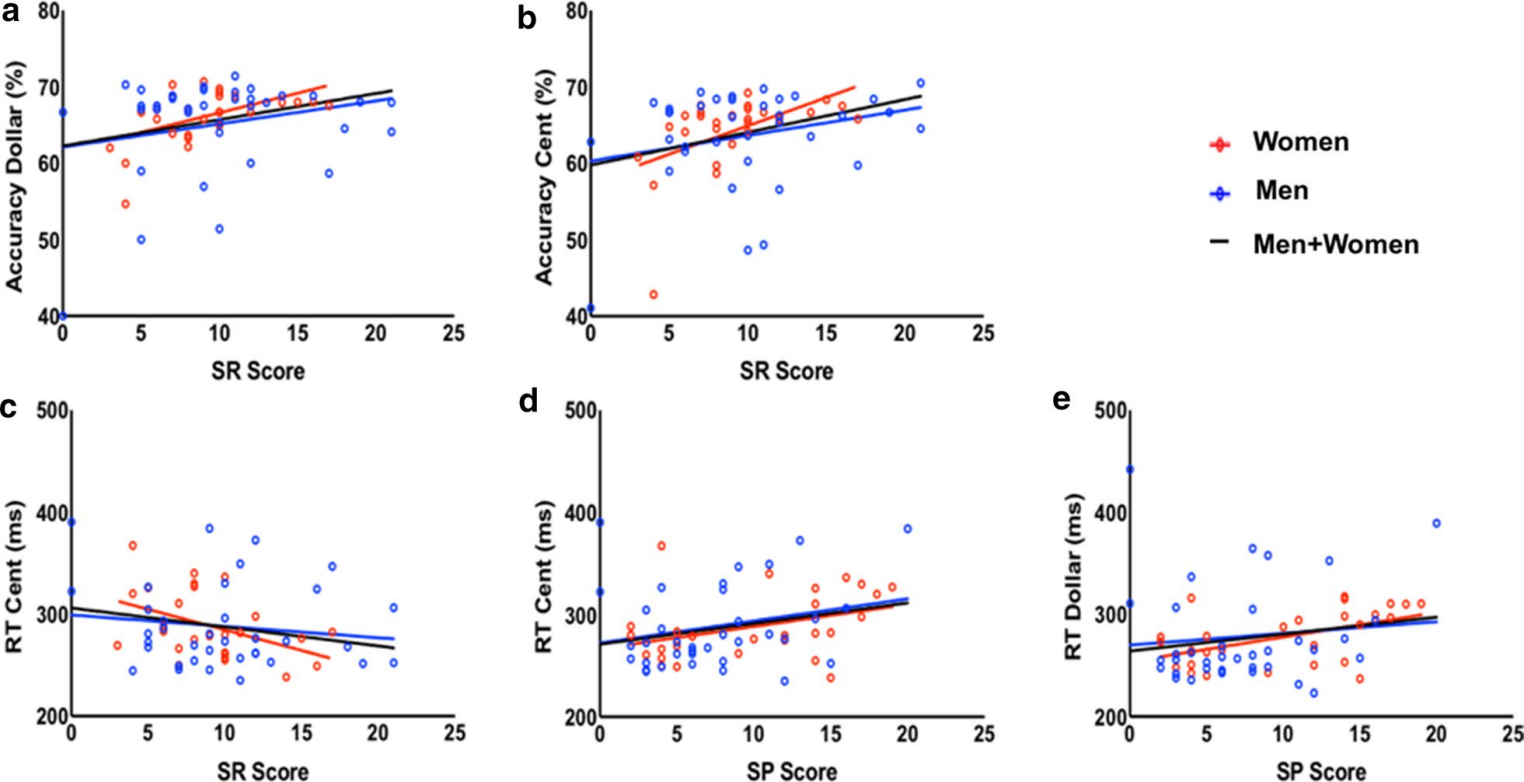
Linear regressions between SPSRQ scores for men, women, and all, for accuracy rate (AR) and RT of dollar and cent trials

Men showed higher sensitivity to reward (SR) score than women, and, in contrast, women showed higher sensitivity to punishment (SP) score than men (Table 1). We examined the relationship between behavioral performance and SR and SP for men and women together and separately. In linear regressions, SR score was positively correlated with the AR of dollar trials in men + women (r_61_ = 0.28, p = 0.02) and in women (r_25_ = 0.52, p = 0.006). SR score was also positively correlated with the AR of cent trials in men + women (r_61_ = 0.33, p = 0.008) and in women (r_25_ = 0.51, p = 0.007). SR score was negatively correlated with the RT of cent trials in women (r_25_ =  − 0.44, p = 0.02), SP score was positively correlated with the RT of dollar trials in women (r_25_ = 0.50, p = 0.007), and SP was positively correlated with the RT of cent trials in men + women (r_61_ = 0.28, p = 0.03) (Fig. 2). The r and p values of all correlations are shown in Table 2. However, although women appeared to show more significant correlations between SR/SP scores and performance measures, relative to men, the slope tests did not reveal significant sex differences in any of these correlations (all p’s ≥ 0.47).

**Table 2:** Linear regressions between performance measures and SR/SP

	AR dollar (%)		AR cent (%)		RT dollar (ms)		RT cent (ms)	
	r	p	r	p	r	p	r	p
SR (M + F)	0.28	0.02*	0.33	0.008*	− 0.19	0.12	− 0.22	0.08
SR (M)	0.24	0.17	0.27	0.12	− 0.17	0.33	− 0.14	0.42
SR (F)	0.52	0.006*	0.51	0.007*	− 0.31	0.11	− 0.44	0.02*
SP (M + F)	0.04	0.74	0.01	0.92	0.21	0.09	0.28	0.03*
SP (M)	0.08	0.65	− 8.47e−4	0.99	0.10	0.53	0.24	0.15
SP (F)	− 0.10	0.62	0.001	0.99	0.50	0.007*	0.37	0.06

Accuracy rate (AR, %) and reaction time (RT, ms) and sensitivity to reward (SR) and sensitivity to punishment (SP) scores for men and women combined (M + F), men (M), and women (F). *p < 0.05. Degrees of freedom for M + F: 61; M: 34; F: 25

### Sex differences in regional responses to reward anticipation (“bet”)

**Fig. 3:**
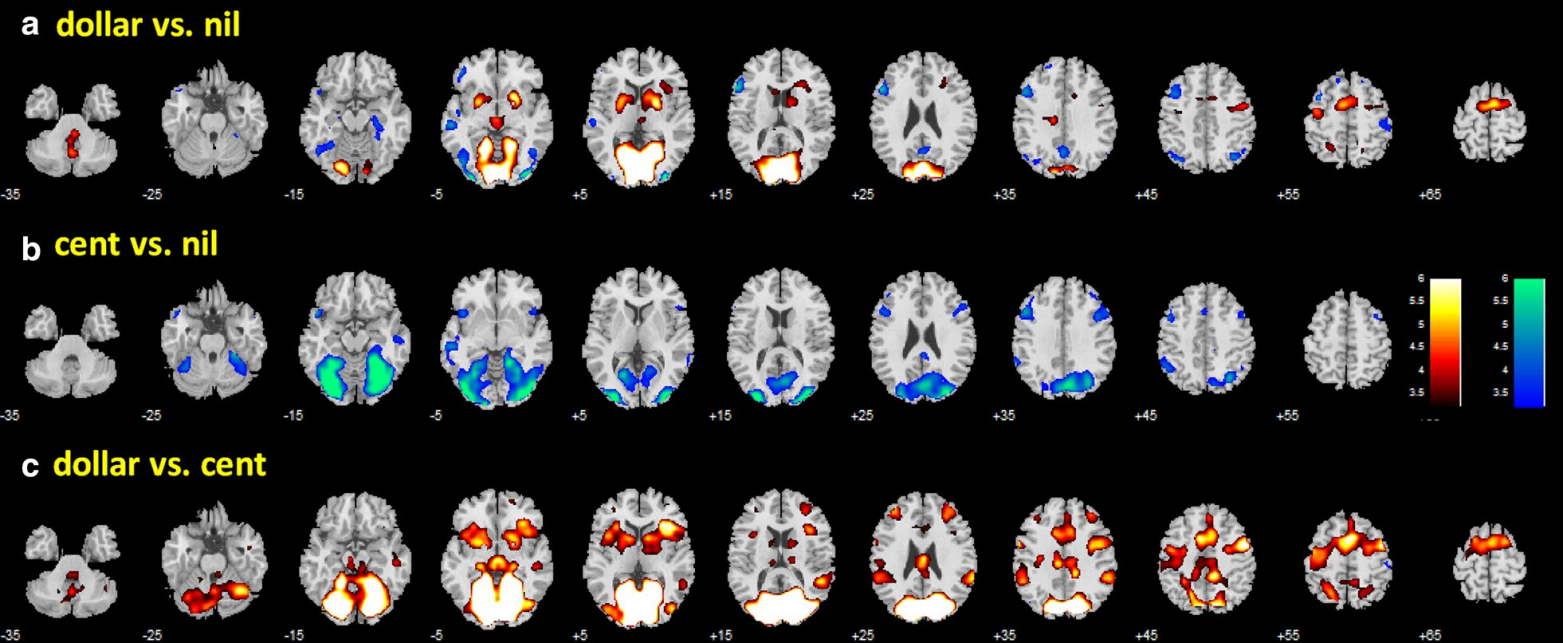
Regional activations during anticipation to win a dollar vs. nil; b cent vs. nil; and c dollar vs. cent. Clusters showing greater response to dollar > nil and nil > dollar, etc., are shown in warm and cool colors, respectively. Color bars indicate voxel T value. Voxel p < 0.001, uncorrected. Neurological orientation: right is right

In a one-sample t test, we evaluated regional activations to anticipation to win dollar vs. nil, cent vs. nil and dollar vs. cent in men and women combined (Fig. 3). Anticipation of a dollar reward involved activation of the medial visual cortical areas, including the cuneus, dorsal striatum, supplementary motor areas, thalamus, and cerebellum. In contrast, anticipation of a cent reward involved “deactivation” of a large swath of medial and lateral visual cortical areas, bilateral inferior frontal cortex, and cerebellum. In a covariance analysis with age and RT differences as covariates, men and women did not show significant differences in activation to reward anticipation during dollar vs. nil, cent vs. nil, or dollar vs. cent trials.

### Sex differences in regional activations to feedback (win or loss)

**Fig. 4:**
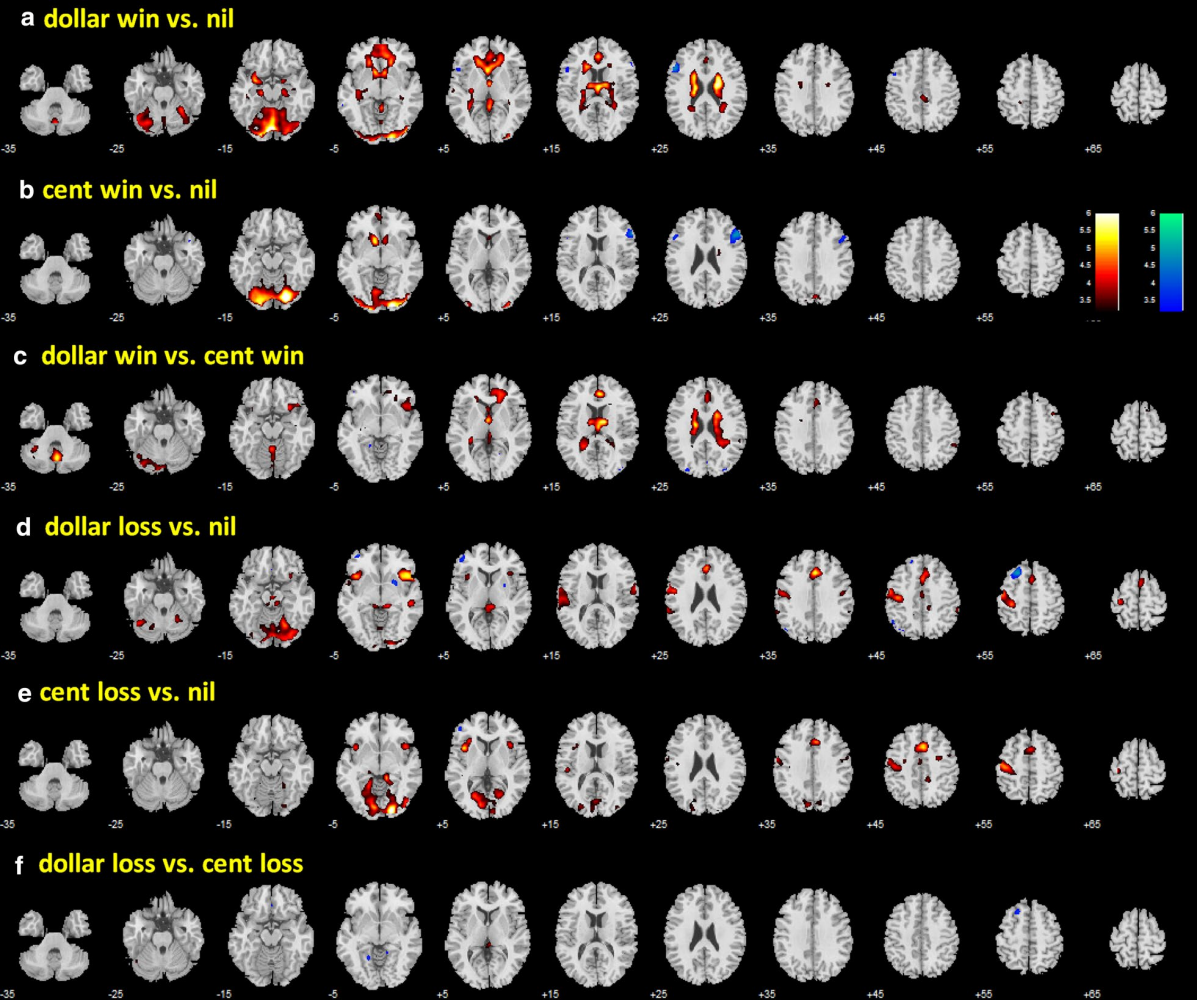
Regional activations to outcomes: a dollar win vs. nil; b cent win vs. nil; c dollar vs. cent win; d dollar loss vs. nil; e cent loss vs. nil; and f dollar vs. cent loss. Clusters showing greater response to dollar > nil and nil > dollar, etc., are shown in warm and cool colors, respectively. Color bars indicate voxel T value. Voxel p < 0.001, uncorrected. Neurological orientation: right is right

In a one-sample t-test, we evaluated regional activations to dollar win vs. nil, cent win vs. nil, dollar vs. cent win, dollar loss vs. nil, cent loss vs. nil, and dollar vs. cent loss in men and women combined. These results are shown in Fig. 4. Both dollar and cent wins engaged higher activation of the ventral striatum (VS) and visual cortical areas. Dollar wins engaged additional brain regions, including the medial orbitofrontal cortex, caudate nucleus and the cerebellum. In contrast, both dollar and cent losses engaged higher activation of bilateral anterior insula and medial frontal cortex, including the anterior cingulate gyrus, and left somatomotor cortex.

**Fig. 5:**
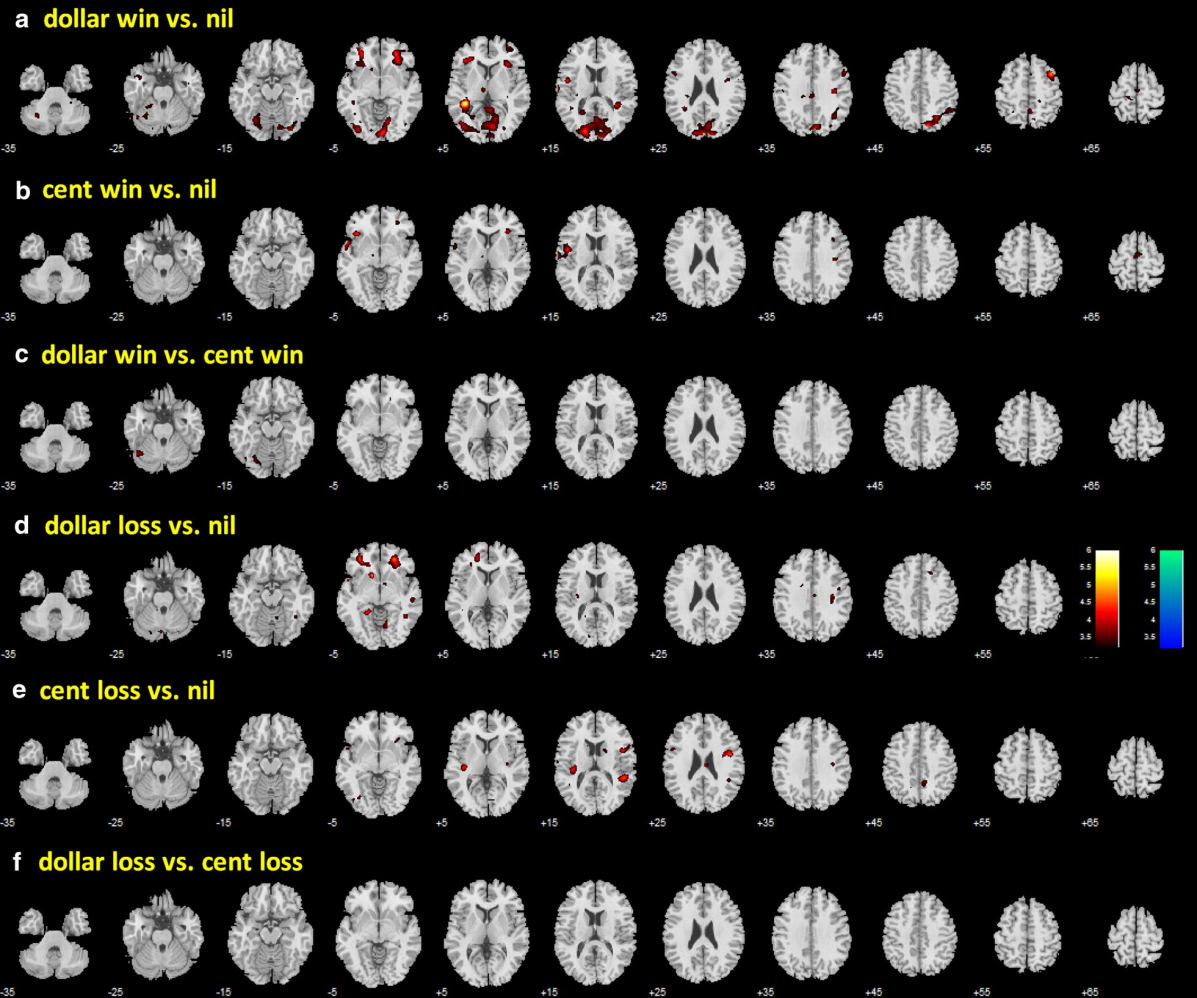
Covariance analyses of men vs. women, with age and RT differences (as specified by the contrast) as covariates, of a dollar win vs. nil, b cent win vs. nil, c dollar win vs. cent win, d dollar loss vs. nil, e cent loss vs. nil, f dollar loss vs. cent loss. Clusters showing greater responses in men vs. women and women vs. men are shown in warm and cool colors, respectively. Color bars indicate voxel T value. Neurological orientation: right is right. Voxel p < 0.001, uncorrected. Clusters that met cluster p < 0.05 FWE corrected are summarized in Table 3

In covariance analyses to compare men and women with age and RT differences as covariates for each of these contrasts, we observed sex differences in activation (men > women) in bilateral orbitofrontal cortex (OFC), and occipital cortex for dollar win > nil (Fig. 5a), and in the left frontal/temporal Rolandic operculum for cent win > nil (Fig. 5b). Compared to women, men also showed higher activation to dollar loss > nil in similar areas of the bilateral OFC and to cent loss > nil in the right precentral gyrus. Clusters meeting cluster p < 0.05 FWE are summarized in Table 3. None of the other contrasts, including dollar win vs. dollar loss and cent win vs. cent loss (not shown in the figure), showed significant sex differences.

**Table 3:** Sex differences (men > women) in regional activations to reward feedback

Cluster size (k)	Voxel (peak Z)	MNI coordinates (mm)			Side	Brain region
		x	y	z		
Dollar Win > Nil						
2497	5.21	− 36	− 40	7	L	Occipital cortex
179	4.45	− 36	32	1	L	Orbitofrontal cortex
246	4.16	27	44	− 5	R	Orbitofrontal cortex
Cent Win > Nil						
196	4.14	− 48	− 4	13	L	Frontal/Temporal Rolandic operculum
Dollar Loss > Nil						
123	4.40	24	44	− 5	R	Orbitofrontal cortex
132	4.14	− 33	44	− 5	L	Orbitofrontal cortex
Cent Loss > Nil						
98	4.31	45	− 1	25	R	Precentral gyrus

Voxel *p* < 0.001 uncorrected; cluster p < 0.05 FWE; R: right; L: left

### Whole brain regression with SP and SR scores

We examined how regional responses to anticipation of dollar vs. nil, cent vs. nil, and dollar vs. cent varied with individual differences in SR and SP. None of the regressions revealed significant findings in men and women combined or alone.

**Fig. 6:**
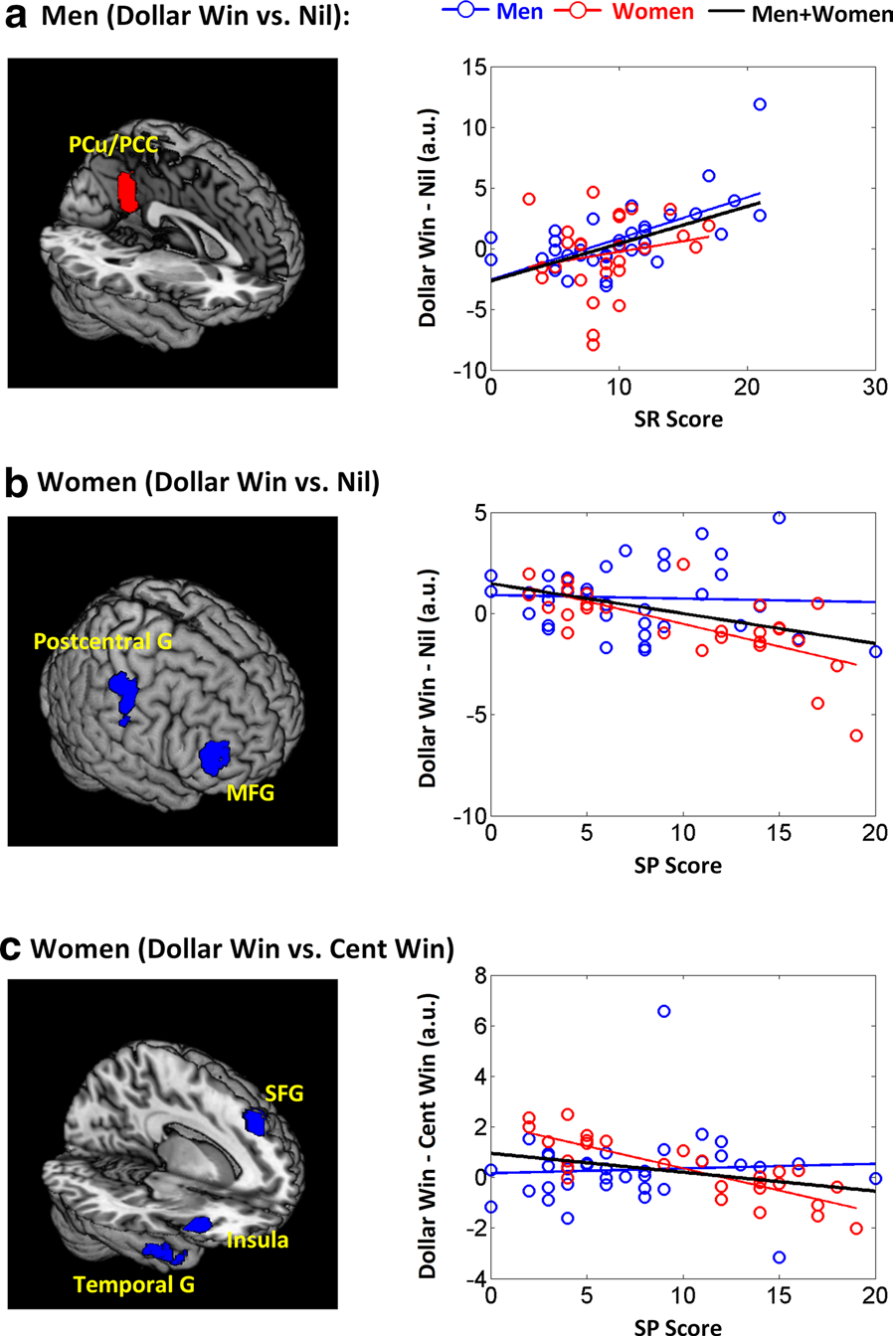
Sex differences in the regression of regional activities with SR/SP scores. The statistics are summarized in the main text. a β contrast “dollar win vs. nil” of the PCC/PCu was positively correlated with SR score in men but not in women; however, the slope test failed to confirm the sex difference (z = 1.88, p = 0.06). b The β contrast “dollar win vs. nil” of the right middle frontal and postcentral gyri was negatively correlated with the SP score in women but not in men, and the slope test confirmed the sex difference in the correlation (z = 3.33, p = 0.0009). c The β contrast “dollar vs. cent win” of the right anterior insula and temporal gyrus and left superior frontal gyrus was negatively correlated with the SP score in women but not in men, and the slope test confirmed the sex difference (z = 4.46, p < 0.0001)

Likewise, we examined how regional responses to feedbacks varied with individual differences in SR and SP. For each of the six contrasts, we performed a linear regression with both SR and SP scores as regressors and years of age and RT difference, as specified by the contrast, as covariates for men and women combined as well as separately. In men alone, the posterior cingulate cortex and precuneus (PCC/PCu) showed activation to dollar win vs. nil in positive correlation with SR score. In women alone, right middle frontal and postcentral gyri showed activation to dollar win vs. nil in negative correlation with SP score. Women also showed activation to dollar vs. cent win in the right anterior insula, left superior frontal gyrus and right temporal gyrus in negative correlation with SP score. These clusters are shown in Fig. 6. Some of these regional activities were also observed in men and women combined. These clusters are summarized in Table 4.

**Table 4:** Regression analysis with SP and SR score

Cluster size (k)	Voxel (peak Z)	MNI coordinates (mm)			Side	Brain region
		x	y	z		
Men + women						
Positive correlation: Dollar Win > Nil vs. SR score						
334	4.66	− 30	17	43	L	Middle frontal G
819	4.51	36	− 28	49	R	Postcentral G
	4.50	6	− 52	28	L/R	PCC/PCu
421	4.23	27	− 40	1	R	Lingual G
Positive correlation: Cent Loss > Nil vs. SR score						
121	4.52	60	− 46	43	R	IPL
162	4.29	− 3	26	49	L/R	dmPFC
104	4.03	33	26	43	R	Middle frontal G
Negative correlation: Dollar Win > Nil vs. SP score						
222	4.50	42	41	13	R	Middle frontal G
Men						
Positive correlation: Dollar Win > Nil vs. SR score						
180	4.19	6	− 52	28	L/R	PCC/PCu
Women						
Negative correlation: Dollar Win > Nil vs. SP score						
268	4.28	45	38	13	R	Middle frontal G
124	4.16	51	− 22	43	R	Postcentral G
Negative correlation: Dollar Win > Cent Win vs. SP score						
105	4.75	36	26	− 11	R	Anterior insula
108	4.24	− 15	38	43	L	SFG
71	4.22	54	5	− 29	R	Temporal G

Voxel *p* < 0.001 uncorrected; cluster p < 0.05 FWE; R: right; L: left; G: gyrus; PCC: posterior cingulate cortex; PCu: precuneus; IPL: inferior parietal lobule; dmPFC: dorsomedial prefrontal cortex; OFC: orbitofrontal cortex; SFG: superior frontal gyrus

To examine sex differences, we performed slope tests [49] on the six individual clusters with regional activities in correlation with the SR or SP score in men or women alone. The results for the individual clusters identified from the same contrast were statistically identical and thus these clusters were combined in slope tests. The results showed that the β contrast “dollar win vs. nil” of the PCC/PCu was positively correlated with SR score in men (r_34_ = 0.665, p = 1.81e-05) but not in women (r_25_ = 0.277, p = 0.180); however, the slope test failed to confirm the sex difference (z = 1.88, p = 0.06). The β contrast “dollar win vs. nil” of the right middle frontal and postcentral gyri was negatively correlated with the SP score in women (r_25_ =  − 0.751, p = 1.53e−05) but not in men (r_34_ =   − 0.080, p = 0.653), and the slope test confirmed the sex difference in the correlation (z = 3.33, p = 0.0009). The β contrast “dollar vs. cent win” of the right anterior insula and temporal gyrus and left superior frontal gyrus was negatively correlated with the SP score in women (r_25_ =   − 0.847, p = 9.24e−08) but not in men (r_34_ =   − 0.024, p = 0.894), and the slope test confirmed the sex difference (z = 4.46, p < 0.0001). The regressions for men and women together and separately are shown in Fig. 6.

## Discussion

We studied sex differences in reward processing in a sample of 63 healthy adults (27 women) using a variant monetary incentive delay task (MIDT). The volunteers made a timed response to win “the bet” of $1, ¢1, or nil. We examined how sex influenced behavioral performance and neural activation to monetary reward anticipation and feedback. Our results show that while there are no sex differences during reward anticipation, male sex is associated with higher activity to feedback of winning $1 (vs. nil) in bilateral orbitofrontal cortex (OFC) and visual cortex, as well as higher activity of losing $1 (vs. nil) in largely the same region of bilateral OFC. Men relative to women also showed higher activation in the left frontal/temporal Rolandic operculum during ¢1 wins vs. nil. These results suggest that men have heightened neural sensitivity to receiving monetary reward of higher or lower magnitude than females and that both wins and losses of large reward were more salient for men, relative to women. As quantified by the SPSRQ, men showed higher sensitivity to reward (SR) and women showed higher sensitivity to punishment, in keeping with the literature [13, 14, 50]. Further, women showed more significant modulation by individual SP in the neural responses to wins and larger wins. We highlight the main findings in the discussion below.

### Lack of sex differences in response to reward anticipation

Our results showed that there were no significant sex differences in neural activation to reward anticipation. Reward anticipation is central to associative learning, and this finding is broadly consistent with animal behavioral studies reporting no sex differences in reward guided associative learning in male and female rodents [27]. In human imaging literature, both men and women have been found to activate a wide neural network to anticipation of monetary reward [38]. Similarly enhanced activation in the VS, ventral tegmental area, and ventromedial prefrontal cortex was observed in both sexes in anticipation of smiling faces of the opposite sex [51]. No sex differences were found in activation of the reward circuits during anticipation of sexually explicit materials [52–54]. However, one study showed increased activation in men vs. women of the ventral putamen during reward anticipation in a gambling task. This same study found that women’s reward circuit was more reactive to anticipation of uncertain reward during the mid-follicular, when estrogen levels are high, than luteal menstrual phase [55]. Thus, our findings need to be interpreted with caution because of a limited sample size and lack of control of menstrual phase in female participants. It is also likely that sex differences or the lack thereof in regional responses to reward anticipation may depend on the behavioral contingencies; i.e., whether the motor decision involves learning or simply guessing or a speedy response to acquire the reward.

### Sex differences in response to feedback and reward/punishment sensitivity

Behaviorally, while men and women each showed higher SR and SP score, in accord with earlier reports using the SPSRQ [14, 56], SP and SR score appeared to influence task performance in both sexes. However, men and women differed in regional responses to reward. Relative to women, men engaged bilateral orbitofrontal cortex (OFC) and visual cortex to a greater extent in response to dollar win vs. nil and the same regions in bilateral OFC in response to dollar loss vs. nil. Men relative to women also showed higher activity in the left frontal/temporal Rolandic operculum to cent win vs. nil. The OFC is involved in learning and optimal decision-making [57], encoding motivational salience of stimuli to support approach behavior [57, 58], and integrating stimulus attributes and emotional value [59–62]. As part of the association cortex, the frontal/temporal Rolandic operculum is implicated in thought, planning and learning of reward contingencies [63–65]. Thus, these findings suggest greater male sensitivity to monetary reward, in accord with the literature. For instance, an event-related potential study in adolescents reported higher feedback P3 amplitude in boys, as compared to girls, in response to monetary rewards [33]. Another study reported a preferential response of the OFC to attractive vs. unattractive faces in men but not in women [66].

Women showed regional responses to wins and larger wins in negative correlation with SP. These areas include the right middle frontal gyrus and anterior insula, each part of the ventral attention and salience networks [67, 68]. In contrast, these regional responses were not correlated with SP scores in men and the responses of these same regions to losses did not correlate significantly with SP scores in women. Thus, higher neural sensitivity to wins appears to be a marker of individual variation in SP in women. Conversely, although falling short of statistical significance (p = 0.06), men seem to demonstrate higher neural sensitivities to wins in relation to SR scores. Thus, reward-related neural responses and behavior may be modulated by distinct personality traits across the two sexes.

In our previous study of the MIDT task, activation of the right caudate head, along with the SMA and right anterior insula during reward anticipation, was correlated with diminished differences in RT collecting a large vs. small reward, suggesting its role in reward-based cognitive motor control [69]. Although one would expect individual SR or SP may be reflected in behavioral performance, we did not observe a significant correlation between the trait and any performance measures in men and women combined or examined separately. More studies with larger sample sizes are needed to revisit this issue.

### Limitations and additional considerations

A number of limitations need to be considered for the study. First, we did not record the menstrual phase of women, which may have introduced variance to influence the current findings. Second, we did not study sex-based variation in general incentive salience. Men relative to women are more motivated by monetary reward; however, the current findings cannot be generalized to other (e.g., social) rewards or behavioral contingencies. Third, the sample size is relatively small to address sex differences. Thus, these findings should be considered preliminary. Finally, the current MIDT variant did not explicitly distinguish sessions where participants were expected to win vs. to lose. Thus, although it is reasonable that participants would expect to win in general (~ 67%) and anticipation did involve regional activities typically implicated in the MIDT, we had no way to evaluate the trial-by-trial variation in the extent participants expected to win or to lose. It is perhaps also for this reason that we did not observe sex differences in anticipation-related activities or an impact of SR and SP traits on these activities. Again, these “negative” results would need to be revaluated with a larger sample and paradigms that instruct anticipation to win vs. lose.

A few additional issues are worth considering. We did not observe a similar pattern of responses (but of different magnitude) to anticipation of dollar and cent wins. In fact, the occipital cortex showed diametric responses between anticipation of dollar and cent wins, suggesting an effect of relativity—large and small reward may not engage the same circuit in a linear fashion. Further, women appear to be more loss aversive than men [70], and it remains to be seen to what extent loss aversion can be captured by punishment sensitivity and how sex differences in loss aversion may account for the current findings. More studies with multiple behavioral paradigms [71] are warranted to investigate these issues.

### Perspectives and significance

Men and women differ in trait and neural sensitivity to reward and punishment. The literature nearly consistently finds men to be more sensitive than women to monetary reward. These heightened neural responses to monetary feedback involve the cognitive motor circuits, including the SMA and caudate nucleus, and would likely influence a wide range of reward-related behavior. The sex differences in reward and punishment sensitivity may have a greater impact on neural activation to reward processing and reward-related decision making than we were able to demonstrate with the data collected of the MIDT. It remains to be seen whether or how these behavioral and neural sensitivity manifest in other laboratory paradigms and real-life decision making. Further, it is unclear how genetic and sociocultural factors may contribute to the sex differences. Sex differences in the incidence and phenomenology of neuropsychiatric and behavioral disturbances such as anxiety, depression, and substance abuse are known to be directly related to reward saliency processing and reward-based learning. It would be of significant interest to public health whether a gender-free or gender-less upbringing, now an emerging trend in child rearing, may alter the picture of sex differences in reward processing and in the susceptibility to neuropsychiatric illness that we find in our society today.

## Conclusions

We replicated higher male sensitivity to reward and female sensitivity to punishment as evaluated by the SPSRQ. Although these individual differences did not translate to influence behavioral performance in the MIDT, likely due to a small sample size, men and women exhibited differences in neural responses to reward. Men show higher SR and neural sensitivity to the receipt of reward, big or small, and to loss, as compared to women. Women showed higher SP than men and individual differences in SP are reflected in regional brain responses to wins and larger wins in women but not in men. These findings add to the imaging literature of sex and individual differences in reward processing.

## Data Availability

The datasets generated and/or analyzed during the current study have not yet been deposited in a public venue, because the study is ongoing. The data analyzed for this study, in raw or processed forms, as well as all analytics are available on request for non-profit academic research. Please contact Dr. Li at chiang-shan.li@yale.edu.

## Abbreviations

AC-PC: Anterior commissure-posterior commissure
AR: Accuracy rate
BOLD: Blood oxygen level-dependent
EEG: Electroencephalography
EPI: Echo-planar imaging
fMRI: Functional magnetic resonance imaging
FRN: Feedback-related negativity
FWE: Family-wise error
HRF: Hemodynamic response function
GLM: General linear model
MNI: Montreal Neurological Institute
MPRAGE: Magnetization-prepared rapid acquisition with gradient echo
MIDT: Monetary Incentive Delay Task
OFC: Orbitofrontal cortex
RT: Reaction time
SMA: Supplementary motor area
SP: Sensitivity to punishment
SPSRQ: Sensitivity to Punishment and Sensitivity to Reward Questionnaire
SR: Sensitivity to reward
SUD: Substance use disorders
TE: Echo time
TR: Repetition time
VS: Ventral striatum (VS)

## References

[1] McClure SM, York MK, Montague PR (2004). The neural substrates of reward processing in humans: the modern role of FMRI. Neuroscientist.

[2] Beck SM, Locke HS, Savine AC, Jimura K, Braver TS (2010). Primary and secondary rewards differentially modulate neural activity dynamics during working memory. PLoS ONE.

[3] Ruff CC, Fehr E (2014). The neurobiology of rewards and values in social decision making. Nat Rev Neurosci.

[4] Baskin-Sommers AR, Foti D (2015). Abnormal reward functioning across substance use disorders and major depressive disorder: Considering reward as a transdiagnostic mechanism. Int J Psychophysiol.

[5] Chau DT, Roth RM, Green AI (2004). The neural circuitry of reward and its relevance to psychiatric disorders. Curr Psychiatry Rep.

[6] Schultz W, Apicella P, Scarnati E, Ljungberg T (1992). Neuronal activity in monkey ventral striatum related to the expectation of reward. J Neurosci.

[7] Schultz W, Tremblay L, Hollerman JR (1998). Reward prediction in primate basal ganglia and frontal cortex. Neuropharmacology.

[8] Haber SN, Knutson B (2010). The reward circuit: linking primate anatomy and human imaging. Neuropsychopharmacology.

[9] Knutson B, Fong GW, Adams CM, Varner JL, Hommer D (2001). Dissociation of reward anticipation and outcome with event-related fMRI. NeuroReport.

[10] Knutson B, Westdorp A, Kaiser E, Hommer D (2000). FMRI visualization of brain activity during a monetary incentive delay task. Neuroimage.

[11] Belin D, Everitt BJ (2008). Cocaine seeking habits depend upon dopamine-dependent serial connectivity linking the ventral with the dorsal striatum. Neuron.

[12] Telzer EH (2016). Dopaminergic reward sensitivity can promote adolescent health: a new perspective on the mechanism of ventral striatum activation. Dev Cogn Neurosci.

[13] Eneva KT, Murray S, O'Garro-Moore J, Yiu A, Alloy LB, Avena NM (2017). Reward and punishment sensitivity and disordered eating behaviors in men and women. J Eat Disord.

[14] Li CR, Huang CY, Lin W, Sun C (2007). Gender differences in punishment and reward sensitivity in a sample of Taiwanese college students. Personal Individ Diff.

[15] Heshmati M, Russo SJ (2015). Anhedonia and the brain reward circuitry in depression. Curr Behav Neurosci Rep.

[16] Volkow ND, Morales M (2015). The brain on drugs: from reward to addiction. Cell.

[17] Hamilton JP, Sacchet MD, Hjornevik T, Chin FT, Shen B, Kampe R (2018). Striatal dopamine deficits predict reductions in striatal functional connectivity in major depression: a concurrent (11)C-raclopride positron emission tomography and functional magnetic resonance imaging investigation. Transl Psychiatry.

[18] Whitton AE, Treadway MT, Pizzagalli DA (2015). Reward processing dysfunction in major depression, bipolar disorder and schizophrenia. Curr Opin Psychiatry.

[19] Kreek MJ, LaForge KS, Butelman E (2002). Pharmacotherapy of addictions. Nat Rev Drug Discov.

[20] Schlaepfer TE, Cohen MX, Frick C, Kosel M, Brodesser D, Axmacher N (2008). Deep brain stimulation to reward circuitry alleviates anhedonia in refractory major depression. Neuropsychopharmacology.

[21] Borsook D, Becerra L, Carlezon WA, Shaw M, Renshaw P, Elman I (2007). Reward-aversion circuitry in analgesia and pain: implications for psychiatric disorders. Eur J Pain.

[22] Robinson TE, Berridge KC (1993). The neural basis of drug craving: an incentive-sensitization theory of addiction. Brain Res Brain Res Rev.

[23] Coffey SF, Gudleski GD, Saladin ME, Brady KT (2003). Impulsivity and rapid discounting of delayed hypothetical rewards in cocaine-dependent individuals. Exp Clin Psychopharmacol.

[24] Giordano LA, Bickel WK, Loewenstein G, Jacobs EA, Marsch L, Badger GJ (2002). Mild opioid deprivation increases the degree that opioid-dependent outpatients discount delayed heroin and money. Psychopharmacology.

[25] Muller KU, Mennigen E, Ripke S, Banaschewski T, Barker GJ, Buchel C (2013). Altered reward processing in adolescents with prenatal exposure to maternal cigarette smoking. JAMA Psychiatry.

[26] Jancke L (2018). Sex/gender differences in cognition, neurophysiology, and neuroanatomy. F1000Res.

[27] Chowdhury TG, Wallin-Miller KG, Rear AA, Park J, Diaz V, Simon NW (2019). Sex differences in reward- and punishment-guided actions. Cogn Affect Behav Neurosci.

[28] Silverman IW (2003). Gender differences in delay of gratification: a meta-analysis. Sex Roles J Res.

[29] Byrnes JP, Miller DC, Schafer WD (1999). Gender differences in risk taking: a meta-analysis. Psychol Bull.

[30] Bjorklund DF, Kipp K (1996). Parental investment theory and gender differences in the evolution of inhibition mechanisms. Psychol Bull.

[31] Evans KL, Hampson E (2015). Sex-dependent effects on tasks assessing reinforcement learning and interference inhibition. Front Psychol.

[32] Ding Y, Wang E, Zou Y, Song Y, Xiao X, Huang W (2017). Gender differences in reward and punishment for monetary and social feedback in children: an ERP study. PLoS ONE.

[33] Greimel E, Bakos S, Landes I, Tollner T, Bartling J, Kohls G (2018). Sex differences in the neural underpinnings of social and monetary incentive processing during adolescence. Cogn Affect Behav Neurosci.

[34] Sheynin J, Beck KD, Pang KC, Servatius RJ, Shikari S, Ostovich J (2014). Behaviourally inhibited temperament and female sex, two vulnerability factors for anxiety disorders, facilitate conditioned avoidance (also) in humans. Behav Processes.

[35] Bobzean SA, DeNobrega AK, Perrotti LI (2014). Sex differences in the neurobiology of drug addiction. Exp Neurol.

[36] Strahler J, Kruse O, Wehrum-Osinsky S, Klucken T, Stark R (2018). Neural correlates of gender differences in distractibility by sexual stimuli. Neuroimage.

[37] Alarcon G, Cservenka A, Nagel BJ (2017). Adolescent neural response to reward is related to participant sex and task motivation. Brain Cogn.

[38] Spreckelmeyer KN, Krach S, Kohls G, Rademacher L, Irmak A, Konrad K (2009). Anticipation of monetary and social reward differently activates mesolimbic brain structures in men and women. Soc Cogn Affect Neurosci.

[39] Le TM, Wang W, Zhornitsky S, Dhingra I, Zhang S, Li CR (2019). Reward sensitivity and electrodermal responses to actions and outcomes in a go/no-go task. PLoS ONE.

[40] Dumais KM, Chernyak S, Nickerson LD, Janes AC (2018). Sex differences in default mode and dorsal attention network engagement. PLoS ONE.

[41] Costumero V, Barros-Loscertales A, Bustamante JC, Ventura-Campos N, Fuentes P, Rosell-Negre P (2013). Reward sensitivity is associated with brain activity during erotic stimulus processing. PLoS ONE.

[42] Kim SH, Yoon H, Kim H, Hamann S (2015). Individual differences in sensitivity to reward and punishment and neural activity during reward and avoidance learning. Soc Cogn Affect Neurosci.

[43] Capa RL, Bouquet CA (2018). Individual differences in reward sensitivity modulate the distinctive effects of conscious and unconscious rewards on executive performance. Front Psychol.

[44] He Z, Zhang D, Muhlert N, Elliott R (2019). Neural substrates for anticipation and consumption of social and monetary incentives in depression. Soc Cogn Affect Neurosci.

[45] Torrubia R, Avila C, Molto J, Caseras X, Molto J, Ohoo X (2001). The sensitivity to punishment and sensitivity to reward questionnaire (SPSRQ) as a measure of Gray's anxiety and impulsivity dimensions. Personal Individ Diff.

[46] Ashburner J, Friston KJ (1999). Nonlinear spatial normalization using basis functions. Hum Brain Mapp.

[47] Friston KJ, Ashburner J, Frith CD, Poline JB, Heather JD, Frackowiak RSJ (1995). Spatial registration and normalization of images. Human Brain Mapping..

[48] Friston KJ, Holmes AP, Poline JB, Grasby PJ, Williams SC, Frackowiak RS (1995). Analysis of fMRI time-series revisited. Neuroimage.

[49] Zar JH (1999). Biostatistical Analysis.

[50] Cross CP, Copping LT, Campbell A (2011). Sex differences in impulsivity: a meta-analysis. Psychol Bull.

[51] Spreckelmeyer KN, Rademacher L, Paulus FM, Grunder G (2013). Neural activation during anticipation of opposite-sex and same-sex faces in heterosexual men and women. Neuroimage.

[52] Stark R, Klein S, Kruse O, Weygandt M, Leufgens LK, Schweckendiek J (2019). No Sex Difference Found: Cues of Sexual Stimuli Activate the Reward System in both Sexes. Neuroscience.

[53] Rupp HA, Wallen K (2008). Sex differences in response to visual sexual stimuli: a review. Arch Sex Behav.

[54] Poeppl TB, Langguth B, Rupprecht R, Safron A, Bzdok D, Laird AR (2016). The neural basis of sex differences in sexual behavior: a quantitative meta-analysis. Front Neuroendocrinol.

[55] Dreher JC, Schmidt PJ, Kohn P, Furman D, Rubinow D, Berman KF (2007). Menstrual cycle phase modulates reward-related neural function in women. Proc Natl Acad Sci USA.

[56] Castella J, Perez J (2004). Sensitivity to punishment and sensitivity to reward and traffic violations. Accid Anal Prev.

[57] Kennerley SW, Walton ME (2011). Decision making and reward in frontal cortex: complementary evidence from neurophysiological and neuropsychological studies. Behav Neurosci.

[58] Wilson RP, Colizzi M, Bossong MG, Allen P, Kempton M (2018). The neural substrate of reward anticipation in health: a meta-analysis of fMRI findings in the monetary incentive delay task. Neuropsychol Rev..

[59] Vollm B, Richardson P, McKie S, Elliott R, Dolan M, Deakin B (2007). Neuronal correlates of reward and loss in Cluster B personality disorders: a functional magnetic resonance imaging study. Psychiatry Res Neuroimaging.

[60] Elliott R, Newman JL, Longe OA, Deakin JF (2003). Differential response patterns in the striatum and orbitofrontal cortex to financial reward in humans: a parametric functional magnetic resonance imaging study. J Neurosci.

[61] Kringelbach ML, O'Doherty J, Rolls ET, Andrews C, Kringelbach ML (2003). Activation of the human orbitofrontal cortex to a liquid food stimulus is correlated with its subjective pleasantness. Cerebral Cortex..

[62] O'Doherty JP (2004). Reward representations and reward-related learning in the human brain: insights from neuroimaging. Curr Opin Neurobiol.

[63] Chase HW, Kumar P, Eickhoff SB, Dombrovski AY (2015). Reinforcement learning models and their neural correlates: an activation likelihood estimation meta-analysis. Cogn Affect Behav Neurosci.

[64] Mălîia MD, Donos C, Barborica A, Popa I, Ciurea J, Cinatti S, Mîndruţă I (2018). Functional mapping and effective connectivity of the human operculum. Cortex.

[65] Worringer B, Langner R, Koch I, Eickhoff SB, Eickhoff CR, Binkofski FC (2019). Common and distinct neural correlates of dual-tasking and task-switching: a meta-analytic review and a neuro-cognitive processing model of human multitasking. Brain Struct Funct.

[66] Cloutier J, Heatherton TF, Whalen PJ, Kelley WM (2008). Are attractive people rewarding? Sex differences in the neural substrates of facial attractiveness. J Cogn Neurosci.

[67] Bernard F, Lemee JM, Mazerand E, Leiber LM, Menei P, Ter Minassian A (2020). The ventral attention network: the mirror of the language network in the right brain hemisphere. J Anat.

[68] Seeley WW (2019). The salience network: a neural system for perceiving and responding to homeostatic demands. J Neurosci.

[69] Dhingra I, Zhang S, Zhornitsky S, Le TM, Wang W, Chao HH, Levy I, Li CR (2020). The effects of age on reward magnitude processing in the monetary incentive delay task. Neuroimage.

[70] Bouchouicha R, Deer L, Eid AG (2019). Gender effects for loss aversion: yes, no, maybe?. J Risk Uncertain.

[71] Li G, Zhang S, Le TM, Tang X, Li CR (2020). Neural responses to reward in a gambling task: sex differences and individual variation in reward-driven impulsivity. Cereb Cortex Commun.

